# Detecting genetic effects on phenotype variability to capture gene-by-environment interactions: a systematic method comparison

**DOI:** 10.1093/g3journal/jkae022

**Published:** 2024-01-30

**Authors:** Xiaopu Zhang, Jordana T Bell

**Affiliations:** Department of Twin Research and Genetic Epidemiology, King's College London, St Thomas’ Hospital, Westminster Bridge Road, London SE1 7EH, UK; Department of Twin Research and Genetic Epidemiology, King's College London, St Thomas’ Hospital, Westminster Bridge Road, London SE1 7EH, UK

**Keywords:** variable quantitative trait loci, vQTL, gene-environment interactions, phenotypic variability

## Abstract

Genetically associated phenotypic variability has been widely observed across organisms and traits, including in humans. Both gene-gene and gene-environment interactions can lead to an increase in genetically associated phenotypic variability. Therefore, detecting the underlying genetic variants, or variance Quantitative Trait Loci (vQTLs), can provide novel insights into complex traits. Established approaches to detect vQTLs apply different methodologies from variance-only approaches to mean-variance joint tests, but a comprehensive comparison of these methods is lacking. Here, we review available methods to detect vQTLs in humans, carry out a simulation study to assess their performance under different biological scenarios of gene-environment interactions, and apply the optimal approaches for vQTL identification to gene expression data. Overall, with a minor allele frequency (MAF) of less than 0.2, the squared residual value linear model (SVLM) and the deviation regression model (DRM) are optimal when the data follow normal and non-normal distributions, respectively. In addition, the Brown–Forsythe (BF) test is one of the optimal methods when the MAF is 0.2 or larger, irrespective of phenotype distribution. Additionally, a larger sample size and more balanced sample distribution in different exposure categories increase the power of BF, SVLM, and DRM. Our results highlight vQTL detection methods that perform optimally under realistic simulation settings and show that their relative performance depends on the phenotype distribution, allele frequency, sample size, and the type of exposure in the interaction model underlying the vQTL.

## Introduction

Genetically associated phenotypic variability has been observed across a broad range of organisms and phenotypes, including for example in human obesity (Wang *et al*. 2019; Yang *et al*. 2012). Two drivers of genetically associated phenotypic variability are gene-gene (GxG) and gene-environment (GxE) interactions. These represent situations in which individuals of a certain genotype can display more divergent phenotypes due to genotype interactions with specific exposures or genetic contexts (Cordell 2002; Romanoski *et al*. 2010; Ashworth *et al.* 2011; Ober and Vercelli 2011; Brown *et al*. 2014; Buil *et al*. 2015; Wang *et al*. 2017, 2019; Viñuela *et al*. 2018; Wang, Fu *et al*. 2014; Bruijning *et al*. 2020). Previous studies have proposed that GxG and GxE interactions commonly influence complex traits including human diseases (Strange *et al*. 2010; Ivarsdottir *et al*. 2017; Wei *et al*. 2017, 2018), animal, and plant breeding outcomes (Hall *et al*. 2007; Jimenez-Gomez *et al*. 2011; Shen *et al*. 2012; Braz *et al*. 2021), and evolutionary traits (Hines *et al*. 2001; Caspi *et al*. 2003; Ansel *et al*. 2008; Feinberg and Irizarry 2010; Kelly *et al*. 2011; Fehrmann *et al*. 2013; Geiler-Samerotte *et al*. 2013; Wolf *et al.* 2015; Xue and Leibler 2018). For example, a GxG interaction between genetic variants in *ERAP1* and *HLA-C* is implicated in susceptibility to psoriasis (Strange *et al*. 2010), and a smoking-genotype interaction in *NAT2* is associated with bladder cancer risk (Vineis *et al*. 2001).

**Fig. 1.**
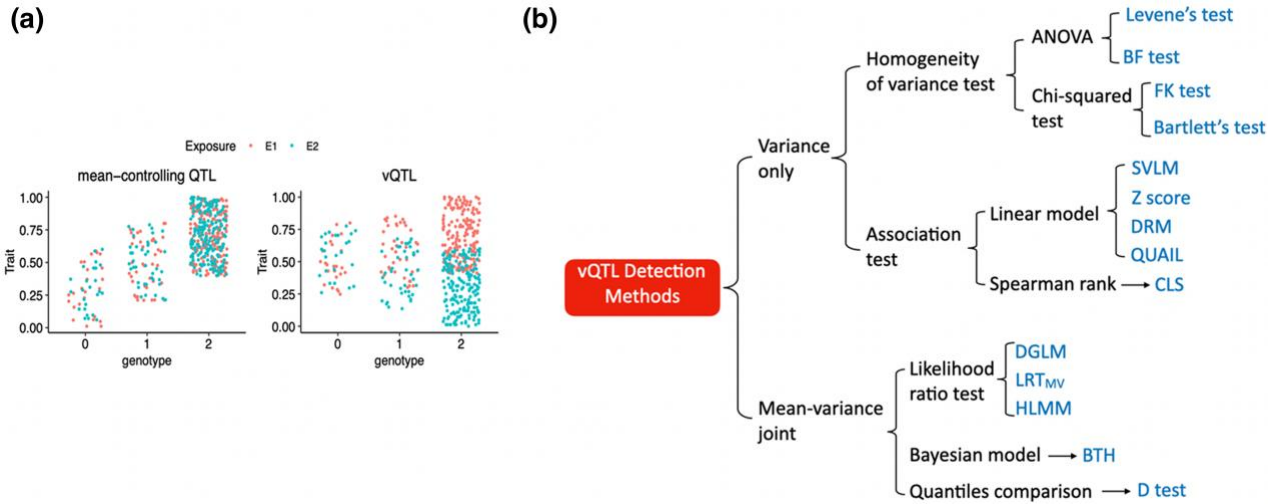
vQTL detection method overview. a) Example of traits associated with a mean-controlling QTL (left) and a vQTL (right). b) Overview of vQTL identification methods. JLS tests that require external annotations of detected QTLs are not included.

Genetic variants associated with phenotypic variability are defined as variable quantitative trait loci, or vQTLs (Fig. 1a). Genetic variants involved in GxG or GxE interactions are one type of vQTLs. Therefore, detecting vQTLs could uncover regulatory mechanisms underlying complex traits. Compared to directly identifying GxG and GxE effects, vQTLs identification is more expedient because it does not require a comprehensive multidimensional search for interactions among multiple factors, or previous knowledge about potential interactions between specific exposures and targeted genetic variants (Paré *et al*. 2010; Romanoski *et al*. 2010; Braz *et al*. 2021).

Multiple vQTL studies have been conducted in humans across a wide range of quantitative and discrete traits (Table 1). Yang *et al*. (2012) discovered a genetic variant in *FTO* to be significantly associated with body mass index (BMI) variability (Yang *et al*. 2012). More recently, Wang *et al*. (2019) identified 75 vQTLs to influence phenotype variability for at least one of nine quantitative traits, including BMI, bone mineral density, and birth weight (Wang *et al*. 2019). Several case-control studies have also identified vQTLs associated with human diseases (Strange *et al*. 2010; Ivarsdottir *et al*. 2017; Wei *et al*. 2017, 2018), for example, rheumatoid arthritis (Wei *et al*. 2017). In terms of intermediate phenotypes, vQTLs in humans have been reported for gene expression (Brown *et al*. 2014; Wang, Yang *et al*. 2014; Sarkar *et al*. 2019), DNA methylation (Ek *et al*. 2018), and protein level variability (Paré *et al*. 2010; Struchalin *et al*. 2010). Further, vQTL findings have also been obtained for multiple biochemical (Revez *et al*. 2020), physical (Conley *et al*. 2018; Marderstein *et al*. 2021), facial (Liu *et al*. 2021), and morphological traits (Córdova-Palomera *et al*. 2021).

**Table 1.** Genome-wide vQTL studies in human samples.

Trait [platform]	Number of samples	Genetic variants	Input data processing	vQTL detection method	Significance threshold	Findings
**Intermediate traits**						
DNA methylation profiles of 431,813 probes (Illumina 450 K) (Ek *et al*. 2018)	729 unrelated blood samples	∼3.4 million SNPs within 500 kb of CpG sites	Rank transformed (Bartlett's) and untransformed (Levene's test) data	Bartlett's and Levene's tests	Bonferroni correction (0.05): *P* < 9.4e-11	590 CpGs were influenced by cis-vmeQTLs, and 14/590 CpGs were affected by GxE. 86% of vmeQTLs were still significant in Levene's test.
Gene expression levels of 35,078 probes (Illumina HT-12 V3) (Wang, Yang *et al*. 2014)	825 adipose, 825 LCL, and 705 skin samples from twins	SNPs within 1Mbp around each probe, ∼1.25 million SNP-probe pairs	Coefficient of variance	FK test and DGLM	*P* < 1e-8	Overall, 99, 75, and 59 are genes influenced by v-eQTL in LCLs, fat, and skin, respectively, and 8 genes are shared across tissues.
Gene expression levels of 13,660 genes (RNA-seq) (Brown *et al*. 2014)	The discovery set: 765 LCL samples from twins The replication set: 462 unrelated LCL samples	SNPs within 1 Mbp around the TSS	Squared residuals after regressing out genotype effects	Spearman's rank correlation between residuals and genotype	FDR (0.05; 5 permutations), and Bonferroni correction (0.05): *P* < 8.9e-10	FDR < 0.05 threshold: 497 v-eQTLs detected and 28/497 v-eQTLs replicated; 181 v-eQTLs are also eQTLs. Bonferroni correction: 23 v-eQTLs were detected, and 16/23 v-eQTLs replicated.
Gene expression levels of 15,574 genes (WG-6 version 1) and 12,076 genes (U133A array), where 10,188 genes were shared (Hulse and Cai 2013)	210 LCL samples from 3 populations	SNPs within 1Mbp around the TSS; 747,524 and 763,745 SNP-probe pairs in the two sets	Coefficient of variance	DGLM and FK test	*P* < 1e-8	166 and 60 genes are associated with at least one v-eQTL respectively in the two datasets, and 8 genes are shared between the two datasets.
Gene expression levels of 9,957 protein-coding genes (single cell RNA-seq) (Sarkar *et al*. 2019)	5,447 iPSCs from 53 unrelated samples of African ancestry	8.47 million SNPs within 100 kb around the TSS	Variance, coefficient of variance, ratio of variance to mean, dispersion parameter	Linear model	Benjamini–Hochberg (0.1)	235 eQTL and 5 v-eQTL detected. 85% of eQTLs are vQTLs; 100% vQTLs are eQTLs.
Protein levels of C-reactive protein and ICAM-1 (High-sensitivity CRP) (Paré *et al*. 2010)	21,799 unrelated blood samples	∼0.34 million SNPs	Log-transformed CRP levels	Levene's test	*P* < 1.5e-7	1 vQTLs for C-reactive protein level variability, and 2 vQTLs for soluble ICAM-1 protein level variability.
**Phenotype traits**						
15 physiological and biochemical traits (Westerman *et al*. 2022)	The discovery set: 254,376 unrelated samples Replication set: 58,927 unrelated samples	∼6.5 million SNPs	Untransformed phenotype values	BF test	Bonferroni correction (0.05): *P* < 4.6e-9	185 vQTLs for 11 traits. 105 of 185 vQTLs show GxE.
20 serum cardiometabolic biomarkers (Lu *et al*. 2022)	The discovery set: 350,016 unrelated samples from four ancestries The replication set: 23,294 unrelated samples	∼9.5 million SNPs	Log-transformed phenotypes and then standardized residuals after covariate adjustment	BF test	Bonferroni correction (0.05): *P* < 4.51e-9	184 vQTL-biomarker pairs at 136 vQTLs were detected; 90.5% vQTLs are mean-controlling QTLs. 27 of 60 candidate pairs replicated. Altogether, 49 of 184 vQTL-biomarker pairs show GxE.
13 physical traits (Wang *et al*. 2019)	348,501 unrelated samples	∼5.55 million SNPs	Adjusted and standardized phenotypes	BF test	*P* < 2e−9	75 vQTLs identified for 9 traits; 66/75 vQTLs are mean-controlling QTLs. Altogether, 16/75 vQTLs show GxE.
BMI, height (Yang *et al*. 2012)	Discovery set: 133,154 unrelated samples Replication set: 36,727 unrelated samples	∼2.68 million SNPs	Rank-based inverse-normal transformed data	Linear model	*P* < 5e-8	6 vQTLs for height and 7 vQTLs for BMI; 1 vQTL for BMI is replicated.
BMI, height (Conley *et al*. 2018)	The discovery set: siblings from 583 families, Replication set: monozygotic and dizygotic twins from the discovery set families	∼0.26 million SNPs	Standard deviation	Linear model	*P* < 1e-5	4 vQTLs for height reach suggestive threshold (*P* < 1e-5), but none replicate. 2 vQTLs for BMI reach suggestive threshold (*P* < 1e-5), and 1 vQTL replicates
BMI (Marderstein *et al*. 2021)	344,201 unrelated samples. 80% in the discovery set, and 20% in the replication set	∼6.7 million SNPs	Dispersion of samples to the median of each group	DRM	*P* < 5e-8	26 vQTLs, 3/26 are mean-controlling QTLs
20 facial traits (Liu *et al*. 2021)	The discovery set: 2,447 unrelated European-ancestry samples Replication set: 5,128 unrelated Asian-ancestry samples	∼3.1 million SNPs	Untransformed phenotype values	BF test	*P* < 5e-8	No signals surpass *P* < 5e-8, but 10 vQTLs reach the *P* < 5e-7 suggestive threshold.
Rheumatoid arthritis (Wei *et al*. 2017)	19,108 unrelated European and US samples (3,323 cases and 15,785 controls)	107,144 autosomal SNPs	Case-control design	BF test	*P* < 5e-8	2 vQTLs detected, and 2 other signals reached suggestive threshold (*P* < 5e-7).
Psoriasis (Wei *et al*. 2018)	Discovery set: European samples, including 2,175 cases and 5,144 controls The replication set: US samples, including 849 cases and 641 controls	Discovery set: ∼5.5 million SNPs Replication set: ∼4.6 million SNPs	Case-control design	BF test	*P* < 5e-8	2 vQTLs were detected in vGWAS, and GWAS. 5 other vQTLs reached a suggestive threshold (*P* < 2.5e-5)
25 hydroxyvitamin D (25OHD) concentration (Revez *et al*. 2020)	318,851 unrelated European-ancestry samples	∼6.09 million SNPs	Normalized adjusted 25OHD level	BF test	*P* < 5e-8	25 vQTLs detected, 23 of them are also mean-controlling QTLs while the remaining 2 are at GWAS suggestive threshold (*P* < 10e-5)
10 brain features (Córdova-Palomera *et al*. 2021)	25,575 unrelated European-ancestry samples	>5 million SNPs	Rank-based inverse normal transformed data	BF test	*P* < 5e-8	1 vQTL detected for mean thickness and thalamus.

LCL, lymphoblastoid cell line; FK, Fligner–Killeen test; DGLM, double generalized linear model; TSS, transcription start site; DRM, deviation regression model; ICAM-1, intercellular adhesion molecule 1; vGWAS, variance genome-wide association studies; GWAS, genome-wide association studies; CRP, c-reactive protein; BMI, body mass index.

A number of methods have been developed to identify vQTLs, and the general premise of these is to detect changes in phenotypic variance across different genotype groups. These methods can be categorized into variance-only and mean-variance joint tests, depending on whether they can detect both mean-controlling QTLs and vQTLs. Variance-only approaches include homogeneity of variance tests (Olkin 1960; Brown and Forsythe 1974; Fligner and Killeen 1976; Conover *et al.* 1981; Li *et al*. 2015), a nonparametric approach (Hong *et al*. 2017), and association tests (Struchalin *et al*. 2012; Yang *et al*. 2012; Brown *et al*. 2014; Topless *et al*. 2015; Marderstein *et al*. 2021; Miao *et al*. 2022) (Fig. 1b). Most mean-variance joint approaches use likelihood ratio tests to assess the role of genetic variants (Smyth 1989; Lee and Nelder 1996, 2006; Rönnegård and Valdar 2011; Cao *et al*. 2014; Young *et al.* 2018). Further methods also include a Bayesian model (Dumitrascu *et al*. 2019) and a quantiles comparison (Aschard *et al*. 2013) (Fig. 1b). Several comparisons of published vQTL detection methods have been carried out, but these have been limited to up to 5 methods to date (Dumitrascu *et al*. 2019; Wang *et al*. 2019; Miao *et al*. 2022;) or certain biological scenarios, for example, when the exposure is discrete (Marderstein *et al*. 2021).

Here, we carry out a simulation study to systematically compare the performance of ten vQTL mapping approaches. We estimated the optimal method under different scenarios considering false positive rate (FPR) and power. The simulation scenarios assume that different types of gene-environment interactions lead to phenotypic variability. We also evaluate the influence of data transformations on the methods' performance and conduct a power analysis. Finally, we apply the optimal methods to validate previously detected vQTLs of human gene expression levels (v-eQTL).

## Materials and methods

### vQTL mapping approaches

Multiple approaches have been proposed to detect variance QTLs, including variance-only, joint mean-variance, and joint location and scale (JLS) tests. Here, we consider these approaches for a systematic comparison in a simulation study design. To clearly describe each method, we assumed that there are N samples collected from altogether G genotype groups. *Y_i_* equals the trait of interest in the *i*th individual, *g_i_* equals the genotype of *i*th individual, and *N_g_* represents the number of samples in *g*th genotype group.

#### Variance-only methods

Tests assessing the heterogeneity of variance are commonly used to detect vQTLs (Fig. 1b). The Levene's test calculates the distance between phenotype data points to the mean phenotypic value of each genotype group as $Z_{gi}=|Y_{gi}-Y_{g}|$ where $Y_{g}$ is the mean value of the *g*th group (Olkin 1960). A one-way ANOVA test was applied of $Z_{gi}$ across *g*th groups, and the generated F statistic approximately follows the F-distribution against *G*–1 and *N*–*G* degree of freedom, where the significance can be estimated (equation 1). Similar to the Levene's test, the Brown–Forsythe (BF) test calculates the distance from each data point to $Y_{g}$, but $Y_{g}$ here is the median value or 10-percent trimmed phenotype mean. The BF test applies a one-way ANOVA to these distances as Levene's test (equation 1). The Levene's test and BF test are implemented in the R package car. Compared to the Levene's test, the BF test was found to be more robust to non-normally distributed data (Brown and Forsythe 1974; Li *et al*. 2015). A generalized Levene's scale test was developed without the requirement of independence of samples in Levene's test (Soave and Sun 2017). It performs the ordinary least squares regression of the trait Y and g and takes the absolute values of the residuals as $Y_{g}$ in Levene's test (equation 1).

The Bartlett's test can also test the null hypothesis that the variances of *G* groups are identical. The Bartlett's test assumes that the distribution of all samples follows a Chi-squared distribution with *N*–1 degree of freedom, and it is a modification of the likelihood ratio test of the approximation of $Chi_{N-1}^{2}$. The Bartlett's test is carried out as described in equation 2, where the pooled variance is $s_{p}^{2}=\overset{G}{\underset{g=1}{\sum }}\;(N_{g}-1)S_{i}^{2}/(N-G)$, and $s_{g}^{2}$ is the variance of the *g*th group. It has been implemented in bartlett.test function in *R*. The Fligner–Killeen (FK) test (Fligner and Killeen 1976) applies a Chi-squared test to the normalized phenotype rankings of samples (equation 3), while the prevalent modified FK test compares rankings of the distance between phenotype data points to the group's median phenotypic value (Conover *et al*. 1981). The FK test ranks all observations based on the $Z_{gi}=|Y_{gi}\;\text{–}\;Y_{g}|$, where $Y_{g}$ is the median value of *g*th group. The rankings of all observations are transformed by the standard normal quantile function $\Phi^{-1}\left(\frac{1}{2}+\frac{Z_{gi}}{2(N+1)}\right)$. The subsequent test statistic is estimated as equation 3 where $A_{g}$ is the mean of the normalized ranking values for the *g*th group, a is the mean of all the normalized ranking values, and $\sigma^{2}$ is the variance of all normalized rankings. The significance of statistics from FK test can be estimated from Chi-squared distribution with *G*–1 degree of freedom. The FK test can be implemented with fligner.test function in R. However, although these methods have been used to detect vQTLs, they also have limitations in that they cannot be used on posterior genotype probabilities and cannot directly account for covariates.

Linear models have also been used to detect vQTLs, and these can take covariates into account (Fig. 1b). The squared residual value linear model (SVLM) first regresses out the genetic effect on the trait mean using the linear model *Y* ∼ *g* (equation 4), and then takes the squared residuals. The squared residuals and genotype are then fit into another linear model, where the significance of *g* determines if the genotype is estimated as a vQTL (Struchalin *et al*. 2012). Similarly, Brown *et al*. (2014) first adjust the phenotype data for genetic effects and then evaluate the Spearman rank correlation between the squared residuals and genotype (CLS, equation 5) (Brown *et al*. 2014). The deviation regression model (DRM, equation 6) (Marderstein *et al*. 2021) and *Z* score method (equation 7) take the distance between phenotype data points to the group's phenotype median and squared *Z* scores generated from inverse normal transformation as response variables (Yang *et al*. 2012; Topless *et al*. 2015), respectively. The quantile integral linear model (QUAIL) establishes quantile regression models. It assumes that the coefficients of linear models between the genotype and different quantile levels will be different when the genetic variant manifests as a vQTL (Miao *et al*. 2022). Specifically, for genotype group *G* in quantile levels, *j*, QUAIL establishes the quantile regression model for the quantile level (equation 8). The accumulated $\beta_{j}$ across different quantiles is then used to estimate the accumulated vQTL effects on trait as $\beta =\overset{0.5}{\underset{0}{\int }}\;(\beta_{1-\tau }-\beta_{\tau })d\tau$. The genetic variant is a vQTL if *β* is greater than 0.


(1) $$\mathrm{Leven}e^{\prime }s\;\mathrm{test}/\mathrm{BF}\;\mathrm{test}:\;F=\frac{N-G}{G-1}\times \frac{\sum_{g=1}^{G}N_{g}\times {\left(Z_{g}-Z_{..}\right)}^{2}}{\sum_{g=1}^{G}\sum_{i=1}^{N_{g}}{\left(Z_{gi}-Z_{g}\right)}^{2}}$$

(2) $$\mathrm{Bartlet}t^{\prime }s\;\mathrm{test}:{\;\mathrm{Chi}}_{N-1}^{2}=\frac{(N-G)\ln\;s_{p}^{2}-\sum_{g=1}^{G}(N_{g}-1)\ln\;s_{g}^{2}}{1+(1/(3(G-1)))\left(\left(\sum_{g=1}^{G}1/(N_{g}-1)\right)-1/(N-G)\right)}$$

(3) $$\mathrm{FK}\;\mathrm{test}:={\mathrm{Chi}}_{G-1}^{2}=\frac{\sum_{g=1}^{G}N_{g}{\left(A_{g}-a\right)}^{2}}{\sigma^{2}}$$

(4) $$\mathrm{SVLM}:\;\mathrm{residual}s^{2}(Y∼g)∼g$$

(5) $$\mathrm{CLS}:\;\;\mathrm{spearman}(\mathrm{residual}s^{2}(Y∼g),g$$

(6) $$\mathrm{DRM}:\;|Y_{gi}-Y_{g}(\mathrm{median})|∼g$$

(7) $$Z\mathrm{score}:\;\Phi^{-1}\;{\left(Y\right)}^{2}∼g$$

(8) $$\mathrm{QUAIL}:\;Q_{Y}\;(\tau |G=g)=\beta_{j}g$$

#### Mean-variance joint tests

The double generalized linear model (DGLM) iteratively fits two generalized linear mixed models (GLM) which model genetic effects on the phenotypic mean and dispersion, respectively, until convergence (Smyth 1989; Rönnegård and Valdar 2011) (equation 9.1–9.2). The DGLM method has been implemented in the dglm R package. Replacing the GLM with a hierarchical generalized linear model (HGLM) could relax the assumption of the GLM that errors are independent and normally distributed (Lee and Nelder 1996, 2006). The LRT_MV_ denotes the major allele homozygous, heterozygous, and minor allele homozygous genotype groups of genetic variants *i* as *g_i_*_0_, *g_i_*_1_, and *g_i_*_2_, respectively, and then estimates genetic effects on the trait variance (equation 10.1), mean (equation 10.2), both (equation 10.3), and neither (equation 10.4). The LRT_MV_ calculates the maximum likelihood of the four models to determine the genetic influence (Cao *et al*. 2014). Likewise, the heteroskedastic linear mixed model (HLMM) calculates the likelihood of genetic effects on traits as well. However, HLMM generates the model with one degree of freedom rather than two degrees of freedom as in LRT_MV_, and it uses likelihood to estimate the genetic effects on variance, mean, genetic dominance, all these three effects, and none (equation 11.1–11.5) (Young *et al*. 2018). HLMM is implemented in the python library hlmm.

The Bayesian heteroskedastic linear regression model (BTH) assumes that there are genetic effects on the trait as follows, $Y∼N(\beta_{0}+g_{i}\beta ,\sigma^{2}\alpha^{-g_{i}})$. The scenario where α≠1 represents the case where the phenotypic variance is a function of genetic variants. Therefore, BTH calculate the Bayesian factor (equation 12) to determine whether the genetic variant affects trait variability (Dumitrascu *et al*. 2019). The D test compares the accumulated differences between quantiles of genotype groups (equation 13). The BTH and D methods both require labor-intensive permutations to determine the appropriate significance threshold, so they are not suitable for genome-wide vQTL mapping (Aschard *et al*. 2013).


(9.1) $$\mathrm{DGLM}:\;\mathrm{Genetic}\;\mathrm{effects}\;\mathrm{on}\;\mathrm{mean}:\;Y_{i}=\mu +g_{i}^{T}\alpha +g_{i}^{T}\beta +\varepsilon_{i}\;\varepsilon_{i}∼N(0,\sigma^{2})$$

(9.2) $$\mathrm{DGLM}:\;\mathrm{Genetic}\;\mathrm{effects}\;\mathrm{on}\;\mathrm{variance}:\;Y_{i}=\mu +g_{i}^{T}\alpha +c_{i}^{T}\beta +\varepsilon_{i}\;\varepsilon_{i}∼N(0,g_{i}^{T}\theta +z_{i}^{T}\gamma )\;$$

(10.1) $$\begin{array}{rl} & \mathrm{LR}T_{\mathrm{MV}}:\;\mathrm{Genetic}\;\mathrm{effects}\;\mathrm{on}\;\mathrm{variance}\;\mathrm{only}:\;Y_{i}=\mu +g_{i1}\alpha_{1}+g_{i2}\alpha_{2}\\ & +c_{i}\beta +\varepsilon_{i}\;\varepsilon_{i}∼N(0,\sigma^{2}) \end{array}$$

(10.2) $$\mathrm{LR}T_{\mathrm{MV}}:\;\mathrm{Genetic}\;\mathrm{effects}\;\mathrm{on}\;\mathrm{mean}\;\mathrm{only}:\;Y_{i}=\mu +c_{i}\beta +\varepsilon_{i}\;\varepsilon_{i}∼N(0,\sigma_{g_{i}}^{2})\;$$

(10.3) $$\begin{array}{rl} & \mathrm{LR}T_{\mathrm{MV}}:\;\mathrm{Genetic}\;\mathrm{effects}\;\mathrm{on}\;\mathrm{both}\;\mathrm{mean}\;\mathrm{and}\;\mathrm{variance}:\\ & Y_{i}=\mu +g_{i1}\alpha_{1}+g_{i2}\alpha_{2}+c_{i}\beta +\varepsilon_{i}\;\varepsilon_{i}∼N(0,\sigma_{g_{i}}^{2}) \end{array}\;$$

(10.4) $$\mathrm{LR}T_{\mathrm{MV}}:\;\mathrm{Null}\;\mathrm{model}:\;Y_{i}=\mu +c_{i}\beta +\varepsilon_{i}\;\varepsilon_{i}∼N(0,\sigma^{2})$$

(11.1) $$\begin{array}{rl} & \mathrm{HLMM}:\;\mathrm{Genetic}\;\mathrm{effects}\;\mathrm{on}\;\mathrm{arbitrary}\;\mathrm{mean}\;\mathrm{and}\;\mathrm{variance}\;\\ & Y|g∼N(\mu_{g},\sigma_{g}^{2}) \end{array}$$

(11.2) $$\begin{array}{rl} & \mathrm{HLMM}:\;\mathrm{Genetic}\;\mathrm{effects}\;\mathrm{on}\;\log\text{-}\mathrm{variance},\;\mathrm{mean},\\ & \;\mathrm{and}\;\mathrm{genetic}\;\mathrm{dominance}\;Y|g∼N(\mu_{g},\exp\;(\mu_{v}+\alpha_{v}g)) \end{array}$$

(11.3) $$\mathrm{HLMM}:\;\mathrm{Genetic}\;\mathrm{effects}\;\mathrm{on}\;\log\text{-}\mathrm{variance}\;\mathrm{and}\;\mathrm{mean}\;Y|g\;∼N(\mu +\alpha g,\exp\;(\mu_{v}+\alpha_{v}g))$$

(11.4) $$\mathrm{HLMM}:\;\mathrm{Genetic}\;\mathrm{effects}\;\mathrm{on}\;\mathrm{mean}\;\mathrm{only}\;Y|g∼N(\mu +\alpha g,\;\sigma^{2})$$

(11.5) $$\mathrm{HLMM}:\;\mathrm{Null}\;\mathrm{model}\;Y|g∼N(\mu ,\sigma^{2})$$

(12) $$\mathrm{BTH}:\;\mathrm{Bayesian}\;\mathrm{factor}:\;\frac{P(Y|g,\alpha \neq 1)}{P(Y|g,\alpha =1)}$$

(13) $$D\;\mathrm{test}:S_{g_{0}↔g_{1}}\sum (q_{g0}^{\mathrm{quantile}}-q_{g1}^{\mathrm{quantile}}{)}^{2}$$

#### Joint location and scale tests

Joint location and scale tests combine tests of genetic effects on the mean (for example, linear model) and variance (for example, Levene's test) to determine whether a genetic variant is associated with a phenotype (Soave *et al*. 2015; Hong *et al*. 2017; Staley *et al*. 2022). However, JLS results can not specify whether the detected signals act as mean-controlling QTLs or vQTLs. Additional tests are required to interpret the results from JLS (Staley *et al*. 2022) in terms of the specific impact on the trait distribution, and therefore JLS tests were not considered in the current simulation design.

In this study, we focus on methods that are suitable for genome-wide vQTL mapping without either time-consuming permutations or extra needs to specify whether the identified genetic variants are vQTLs or mean-controlling QTLs. Therefore, we do not consider the above-mentioned BTH, D test, and JLS tests in the comparative simulation analysis. We assume all samples are unrelated, so we also exclude the generalized Levene's scale test. Furthermore, the original LRT_MV_ code is not available and since its performance was proposed to be similar to that of the DGLM (Cao *et al*. 2014), it is excluded from our comparison as well. Lastly, we do not consider HLMM either because we observed that small numerical values can lead to the model failing to converge during testing, and the majority of simulated QTL effects here are small, reflecting published findings. Altogether, this study carries out a simulation-based comparison of 10 vQTL mapping methods, including Levene's test, BF test, FK test, Bartlett's test, SVLM, DRM, Z score, CLS, QUAIL, and DGLM.

### Simulation design

We assume that a quantitative phenotype is under the influence of additive genetic effects at a single locus (G), environmental effects from a single exposure (E), GxE interaction effects, and noise (equation 14). We assume that the genetic variant that interacts with environmental factor is the common allele at the single biallelic locus. The exposures E that contribute to the GxE term can be either discrete (e.g. a binary exposure) or continuous (E ∼ uniform (20, 70) or E ∼ normal (25, 3)). To further explore whether the genetically associated environmental factor will influence the methods' performance, we assumed that the genetically associated environmental factor E1 is defined as *E*1 = *E* + *a_mean_G*. Errors are generated from the normal, Chi-squared (*df* = 1), and Gamma (shape1 = 2, shape2 = 0.5) distributions. We rescaled each term (*G*, *E*, G×E, and error) from 0 to 1 with min–max normalization, then we generated the trait Y ensuring that all coefficients (*a_mean_, a_E_, a_var_,* and *a_error_*) sum to 1.


(14) $$Y=\alpha_{\mathrm{mean}}\times G+\alpha_{E}\times E+\alpha_{\mathrm{var}}\times G\times E+\alpha_{\mathrm{error}}\times \mathrm{error}$$

The total sample size (*N*) and minor allele frequency (MAF) are used to determine the sample size in the 3 genotype groups. To distinguish vQTLs from genetic variants that are involved in other biological scenarios, such as parent-of-origin effects, we require that there are at least 10 samples in each genotype group. To test different biological scenarios, we set *a_error_* at 0.2. The effect sizes are set at *a_mean_, a_var_* = {0, 0.005, 0.01, 0.03, 0.05, 0.1, 0.15, 0.2, 0.25, 0.3, 0.35, 0.4}. Models are evaluated under situations where the genetic variant impacts, (1) The phenotype level only (*a_var_* = 0, *a_mean_* ≠ 0), (2) The phenotype variance only (*a_mean_* = 0, *a_var_* ≠ 0), (3) Both phenotype level and variance (*a_mean_* = 0.1, *a_var_* ≠ 0), or (4) Neither phenotype level nor variance (*a_var_* = *a_mean_* = 0).

Changes in phenotype variance across groups could lead to differences in the mean phenotype level, which is a hurdle to establishing a variance-only situation. Specifically, if we assume that a trait is influenced by a GxE interaction only, with a single genetic risk variant at a single locus, then there are *N*_1_, *N*_2_, and *N*_3_ unrelated samples in the minor homozygote, heterozygote, and major homozygote group, respectively. Allele *G* at this locus interacts with exposures which can be classified into *k* classes (*E*_1_, *E*_2_, …, and *E_k_*). The frequencies of samples that are exposed in each exposure are *f*_1_, *f*_2_, …, and *f_k_*. The mean phenotypic level of the trait in each genotype group is in equation 15.


(15) $$\left(\overset{k}{\underset{i=1}{\sum }}\;G\times E_{i}\times f_{i}\times N\right)/N=\overset{k}{\underset{i=1}{\sum }}\;G\times E_{i}\times f_{i}$$

Therefore, the expected mean phenotype levels of the major homozygote, heterozygote, and minor homozygote groups are $\overset{k}{\underset{i=1}{\sum }}\;E_{i}\times f_{i}$, $\overset{k}{\underset{i=1}{\sum }}\;E_{i}\times f_{i}$, and 0, respectively. The mean phenotype value is influenced by the sample proportions across exposures (*f_i_*) and by the encoded exposures (*E_i_*). Therefore, a *G×E* interaction could lead to the shift of the mean of each genotype group. To design a variance-only situation, we need to consider sample proportions across exposure categories and how to encode exposures. For example, when the exposure is binary (*E* and *e*) and when *f_E_* is 10% and *f_e_* is 90%, E and e would need to be encoded as 9 and −1 to ensure that there is no difference in the mean phenotype value among the three genotype groups. To simplify our simulation design, we only estimate variance-only situations when the exposure is binary.

We also estimate the power of the methods across a set of parameters, including sample size [*N* = (500, 1,000, 5,000, 10,000, 20,000, 50,000, 100,000, 200,000)] and MAF = (0.05, 0.1, 0.2, 0.3), and different frequency of samples in the two exposure categories when the exposure is binary [f_E_ = (0.1, 0.3, 0.5)].

### Data preprocessing and method performance assessment

When detecting QTLs, it is often necessary to normalize the phenotype as well as to adjust the phenotype for multiple biological covariates, for example, age, sex, and smoking, as well as technical covariates such as processing batch. Therefore, we adjusted the phenotype for multiple covariates, including the main effect of the hypothesized environmental exposure (*E*) before vQTL detection, as detailed below. For SVLM and CLS, we used a linear model to adjust phenotype levels on both *E* and genotype (equation 16.1). For the remaining eight methods, we used a linear model to adjust phenotype levels on *E* (equation 16.2). The resulting residuals and genotype data were then input into each of the 10 tested methods to evaluate method performance. To assess the performance of each of the 10 tested methods, we estimated the frequency of significant genetic effects (*P* ≤ 0.05) detected by the method for 1,000 simulations. The coefficients of the genotype term in DGLM, SVLM, DRM, QUAIL, and *Z* score were used as the estimated effect size.


(16.1) Y∼G+E

(16.2) Y∼E

The interacting environmental factor may be not observed in real scenarios. Therefore, we also estimated the methods' performance without first regressing out *E* as a covariate. Therefore, in this setting, the input data Y to each method are the raw data.

Some methods are also sensitive to the distribution of the phenotype, specifically, Levene's test, and normalizing the phenotype data (or phenotype residuals after covariate adjustment) may improve model performance (Yang *et al*. 2012; Córdova-Palomera *et al*. 2021). Therefore, we also used the normalized residuals as input data for all methods, except for the *Z* score method which already applies a rank-based inverse normal transformation.

### Model application in real data

We applied a subset of the 10 tested methods to detect previously identified variance QTLs in gene expression data (v-eQTLs). To this end, we used the datasets and results from (Brown *et al*. 2014; Wang, Fu *et al*. 2014; Wang, Yang *et al*. 2014) in detecting v-eQTLs in lymphoblastoid cell line (LCL) samples from twins from the TwinsUK cohort. Brown *et al*. (2014) used 765 samples and the gene expression profile was generated from RNA-seq, and (Wang, Fu *et al*. 2014; Wang, Yang *et al*. 2014) used 825 samples and the gene expression data were generated from Illumina Human HT-12 V3 BeadChips. Brown *et al*. (2014) used the CLS method to detect v-eQTLs after correction for family structure, and they conducted a set of 5 permutation tests for multiple testing corrections, observing 508 associations of v-eQTLs-gene pairs (FDR < 0.05). Wang, Fu *et al*. (2014) (Wang, Yang *et al*. 2014) randomly split twins from the same family into two groups. In one sample group, they applied the FK test and filtered the SNP-probe pairs surpassing *P* < 0.01, and then they used the DGLM method to these filtered SNP-probe pairs to detect v-eQTLs. They conducted 10,000 permutations on each significant SNP-probe pair resulting from DGLM to reduce the FPR. Finally, they validated the significant SNP-probe pairs (*P*_permutation_ < 0.001) in the other twin sample group. Altogether 99 genes were detected and validated to be associated with at least one cis-v-eQTLs in Wang, Fu *et al*. (2014).

In our study, we obtained genotype and gene expression data from LCL samples from 765 samples from the TwinsUK cohort. We obtained the same samples and the same version of genotype imputation and gene expression data as those analysed by Brown *et al*. (2014). We focused on 5 genes that were detected to be associated with 5 v-eQTLs in both previous studies, and 5 v-eQTLs and 3 v-eQTLs that were reported to have epistatic effects in Brown *et al*. (2014) and Wang, Fu *et al*. (2014) (Wang, Yang *et al*. 2014), respectively. We also considered 9 and 3 genetic variants that were reported to be in epistatic interactions with the v-eQTLs (epi-QTLs) in the original publications, respectively. Altogether, we explored 22 genetic variants and gene expression levels at 5 genes in the current study (Table 2).

**Table 2.** Validation of v-eQTLs from Brown *et al*. (2014) and Wang, Fu *et al*. (2014).

Study	Gene	SNP (chr:position)	rsID	Original sample size	Validation sample size	Validation methods			Original methods		
						DRM *P* value	SVLM *P* value	BF *P* value	CLS *P* value	FK *P* value	DGLM *P* value
v-eQTLs											
Brown *et al*	NUDT2	9:34318654	rs10972055	765	765	1.3e-14	2.7e-7	1.6e-13	9.5e-18	2.0e-17	9.3e-30
Brown *et al*	BTN3A2	6:27024687	rs12190473	765	765	2.6e-6	1.6e-4	3.6e-5	**1.3e-7** *a*	2.9e-6	3.5e-5
Brown *et al*	CAPN11	6:44132782	rs3757273	765	765	1.6e-11	6.3e-10	6.8e-11	3.1e-24	4.3e-11	2.7e-29
Brown *et al*	PLXDC2	10:20254725	rs11011705	765	765	**N.S**	**0.04** *^a^*	**N.S**	**N.S**	**N.S**	9.4e-8
Brown *et al*	USP6	17:5097573	rs62072760	765	765	2e-15	8.2e-7	9.7e-11	7.6e-64	9.3e-16	1.6e-148
Wang *et al*	NUDT2	9:34334015	rs1337593	825	765	9.6e-15	2.7e-7	1.1e-13	6.7e-18	1.3e-17	8e-30
Wang *et al*	BTN3A2	6:26415637	rs742090	825	765	3e-3	0.04	3e-3	2e-4	1e-4	0.03
Wang *et al*	CAPN11	6:44141088	rs6938938	825	765	6.6e-31	4.6e-31	6e-33	**3.2e-35** *^a^*	1.8e-33	8.3e-40
Wang *et al*	PLXDC2	10:20207349	rs2038912	825	765	3.8e-21	6.4e-12	1.2e-21	6.7e-49	6.1e-26	7.5e-70
Wang *et al*	USP6	17:5005311	rs6502843	825	765	2.2e-12	1.1e-5	3.9e-10	3.7e-47	1.9e-13	5.7e-114
epi-QTLs											
Brown *et al*	NUDT2	9:34381192	rs3753033	765	765	1.8e-5	1.8e-3	8.5e-4	**7.9e-5** *^a^*	6.4e-5	2e-11
Brown *et al*	NUDT2	9:34256347	rs10814083	765	765	7e-4	9.e-3	0.01	4.8e-4	2e-3	3.2e-7
Brown *et al*	NUDT2	9:34284727	rs7028914	765	765	7.9e-6	1.7e-4	5.7e-5	**4.5e-3** *^a^*	4.7e-5	6.8e-14
Brown *et al*	BTN3A2	6:26276650	rs9393692	765	765	1e-4	5.8e-5	7e-3	**6e-3** *^a^*	**2.1e-3** *^a^*	4.1e-9
Brown *et al*	BTN3A2	6:27261324	rs7746199	765	765	8e-4	**0.04** *^a^*	0.01	**1.7e-3** *^a^*	1.4e-3	2.2e-4
Brown *et al*	CAPN11	6:44136899	rs4711770	765	765	1.6e-5	3.2e-4	1.2e-3	5.9e-8	2.8e-4	1.6e-17
Brown *et al*	PLXDC2	10:20273583	rs1111367	765	765	3.5e-13	4.6e-6	2.9e-11	2.5e-31	1.5e-18	1.2e-25
Brown *et al*	PLXDC2	10:20198883	rs11594747	765	765	**N.S**	**N.S**	**N.S**	**N.S**	**N.S**	**N.S**
Brown *et al*	USP6	17:5213391	rs2585266	765	765	2.2e-33	3.8e-19	1.8e-34	2.4e-50	4.6e-47	Failed to converge
Wang *et al*	NUDT2	9:34167694	rs7032924	825	765	**N.S**	**N.S**	**N.S**	**0.01** *^a^*	**N.S**	**0.02** *^a^*
Wang *et al*	BTN3A2	6:26404374	rs3799378	825	765	3e-4	3.6e-6	0.01	3.2e-7*^a^*	1.6e-4	1.4e-5
Wang *et al*	PLXDC2	10:20238864	rs1326233	825	765	2e-31	3.4e-16	1.6e-34	1.8e-38	8.2e-34	3.6e-47

N.S means the statistic of related methods is not significant.

^*a*^Represents the method fails to surpass the FDR < 0.05.

The gene expression data were corrected for family structure, zygosity, primer index, age, BMI, GC (guanine-cytosine) content, and insert size mode with a linear mixed model. The resulting residuals were then input into CLS and the combination of FK and DGLM to replicate the original findings for v-eQTLs and epi-QTLs. We also applied BF, SVLM, and DRM for the detection of v-eQTLs and epi-QTLs. We carried out 20 permutations to calculates the false discovery rate (FDR) and used FDR < 0.05 to determine the significance of v-eQTLs and epi-QTLs.

## Results

We carried out a systematic comparison of 10 previously proposed methods to detect variance QTLs. These include Levene's test, the BF test, the FK test, Bartlett's test, theSVLM, the DRM, the *Z* score, the correlation least squares (CLS), the QUAIL, and the DGLM. Each method performance was evaluated using FPR and the discovery rate based on simulation results. We evaluated method performance on datasets with different sample sizes (500, 1,000, 5,000, 10,000, 20,000, 50,000, 100,000, and 200,000), MAFs (0.05, 0.1, 0.2, and 0.3), environmental factor distributions (binary, normal, and uniform distributions), and phenotype distributions (normal, Chi-squared, and gamma distributions).

### vQTL detection methods comparison for discrete environmental exposures

We initially assumed that a GxE interaction leads to increased phenotypic variability and that the exposure is discrete, such as smoking (current smokers or not). We set a range of effect sizes of *G×E* interaction effects and errors were generated from normal, gamma, or Chi-squared distributions. Results are presented for a sample size of 1,000 unrelated individuals with a genetic variant at 0.05 MAF, and the sample proportions in the two exposure groups are 90 and 10%.

**Fig. 2.**
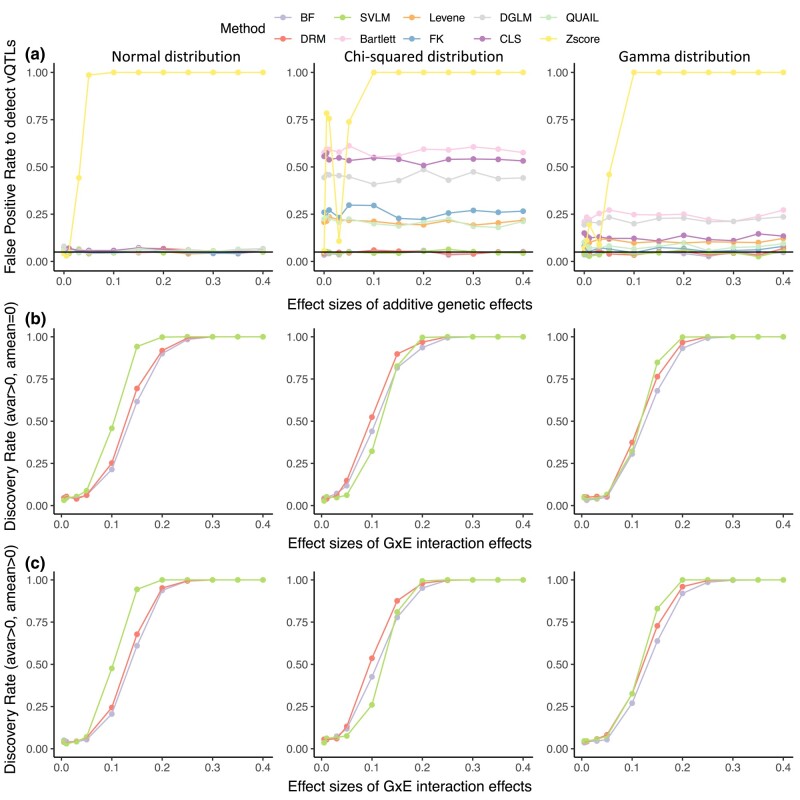
Method performance when the interacting exposure is binary. The discovery rate of methods under the scenario that a single genetic variant affects a) trait level only (*a_mean_* ≥ 0, *a_var_* = 0), which includes the situation where there is no genetic effect (*a_mean_* = 0, *a_var_* = 0), b) trait variance only (*a_mean_* = 0, *a_var_* > 0), and c) trait level and variance (*a_mean_* = 0.1, *a_var_* > 0). The black horizontal line corresponds to a rate of 0.05. The trait is also affected by noise and environmental factors. The first, second, and third columns represent errors generated from normal, Chi-squared, and gamma distributions, respectively.

The Z score method shows high FPR across all error distributions (Fig. 2a). The FPR of the *Z* score method slightly decreases and then increases again when the phenotype follows non-normal distributions. When there are additive genetic effects only, the trait (*Y*) is simulated across the three genotype groups as *a_mean_*× 0 + error (for *G*_0_), *a_mean_* × 1 + error (for *G*_1_), and *a_mean_* × 2 + error (for *G*_2_). Therefore, the ranges of *Y* values are 0 < *Y* < error (for *G*_0_), *a_mean_* < *Y* < *a_mean_* + error (for *G*_1_), and 2*a_mean_* < *Y* < 2*a_mean_* + error (for *G*_2_), respectively. If error > 2*a_mean_* or error < *a_mean_*, the variance of the three genotype groups would appear to be different after normalizing the input data *Y*. Therefore, the mean-controlling QTL will be a spurious signal and the FPR will be high. The FPRs of Bartlett's test, CLS, DGLM, FK, Levene's test, and QUAIL are near 0.05 when the error follows a Normal distribution, but greater when the error is from a non-normal distribution (Fig. 2a). The corresponding FPRs are higher when the error is from the Chi-squared distribution compared to errors from the Gamma distribution. These methods are sensitive to trait skewness because Chi-squared distributed errors lead to the greatest skewness of traits, but normally distributed errors contribute the least to trait skewness. The SVLM, BF, and DRM methods show a consistently low FPR whatever the error distribution (Fig. 2a). These findings are consistent with previous comparisons, which suggest that the FPR is higher for the DGLM and *Z* score regression methods, and lower for the SVLM and DRM methods (Wang *et al*. 2019; Marderstein *et al*. 2021). Our analysis found that QUAIL shows a high FPR, which is inconsistent with the findings from Miao *et al*. (2022) that QUAIL is good at controlling FPRs (Miao *et al*. 2022). When we increase the simulated sample size to 200,000 as in Miao *et al*. (2022) we still observe inflated FPRs for QUAIL. Given that Miao *et al*. (2022) assume that the trait distribution is less skewed (Chi-squared distribution with 6 degree of freedom (df) in Miao *et al*. (2022) vs Chi-squared distribution with 1 df in this study), we also evaluated the FPR of QUAIL under the setting where the trait follows the Chi-squared distribution (*df* = 6) (Miao *et al*. 2022) and observed that the FPR of QUAIL is near 0.05 whatever the sample size is. Therefore, the differences in QUAIL performance are due to differences in the trait distribution, and QUAIL shows inflated FPRs when the trait is mostly skewed (Supplementary Fig. 1).

In terms of the discovery rates, we focused on the three methods—BF, DRM, and SVLM—which do not show inflated FPRs. We observed that the SVLM method performs the best with normally- and gamma-distributed traits (Fig. 2b and c), irrespective of whether there is a mean difference in the phenotypic level across genotype groups. The DRM method is the optimal method when the traits follow Chi-squared distribution.

We then explored the method’s performance with different sample sizes (Supplementary Table 1). For Normally distributed data, the conclusions regarding FPRs and discovery rates are consistent with the previous settings. For MAF of 0.05 with non-normally distributed data, SVLM performs the best when the sample size is 500, but DRM is optimal when the sample size is 1,000 or larger. We hypothesize that this is because an unbalanced sample distribution across exposure categories affects the performance of DRM. The imbalance would lead to many zero values in the three genotype groups and reduce the power of DRM. Specifically, when a single genetic variant (where *G*_0_, *G*_1_, and *G*_2_ represent the three genotype groups encoded as 0, 1, and 2) interacts with a binary exposure (E and e), we assume that 90 and 10% of samples are exposed to *E* and *e* in each genotype group, respectively. Because most samples are exposed to *E*, the median phenotype values for the three genotype groups are 0 (90% of values are *G*_0_ × *E* = 0 and 10% of values are *G*_0_ × *e* = 0), *E* (90% are *G*_1_ × *E* = *E* and 10% *G*_1_ × *e* = *e*), and 2*E* (90% are *G*_2_ × *E* = 2*E* and 10% *G*_2_ × *e* = 2*e*). Therefore, 90% of the distance between the data point to the median value is 0 in each genotype group. Since these distances are the response variables in the linear model in the DRM method, the DRM method has lower power under these particular parameter settings.

We also considered the methods' performance under different MAFs. SVLM and DRM remain the optimal methods with a sample size of 1,000 and MAF of 0.05, 0.1, or 0.2. However, when the MAF is 0.2 or higher, the BF method also performs almost equally well or better than SVLM and DRM, irrespective of data distributions and environmental factor distributions. The reason could be that a higher MAF changes the phenotypic variance of each genotype group. A higher MAF leads to a more balanced sample distribution across the three genotype groups. The input data for each model are the phenotype data residuals after regressing out covariates, which follow the Normal distribution. Therefore, the variance of the phenotype data residuals in the heterozygote genotype class could be smaller than that of either homozygous group (Supplementary Fig. 2 as an example), which would reduce the power of SVLM and DRM as these are based on linear models.

The estimated effect sizes for vQTLs measure to what extent the genetic variants influence phenotypic variability, which may be assessed differently across the different methods (Supplementary Fig. 3). The estimation of effect sizes can be useful for downstream analyses and interpretation, and of the optimally performing methods both SVLM and DRM estimate effect sizes, while BF does not. However, the interpretation of estimated effect sizes is different across methods. For example, the effect size estimated from DRM is the allele effect on the distance from the sample's phenotypic value to the genotype group's median phenotypic value, while SVLM estimates the allele effect on the squared residuals of phenotypic value.

In summary, in the presence of a *G×E* interaction with a discrete exposure, the SVLM, with a MAF less than 0.2, SVLM is optimal with normally distributed data. The optimal model changes from SVLM to DRM when the sample size increases when the data are non-normally distributed. BF is one of the optimal methods when MAF is 0.2 or greater, irrespective of data distribution.

### vQTL detection methods comparison for continuous environmental exposures

We next considered GxE interactions that lead to phenotypic variability where the exposure is continuous, such as BMI. We estimated the performance of the 10 methods with continuous exposures that follow the normal or uniform distributions. Similar to the methods comparison with discrete exposures, we set the same range of effect sizes for *G×E* interactions and the errors are again generated from normal, Chi-squared, or gamma distributions. The results are obtained for a sample size of 1,000 unrelated individuals with a genetic variant at 0.05 MAF. Unlike the discrete exposure scenario where we controlled the sample proportions in the different exposure groups, here we did not control the sample proportions for continuous exposures.

**Fig. 3.**
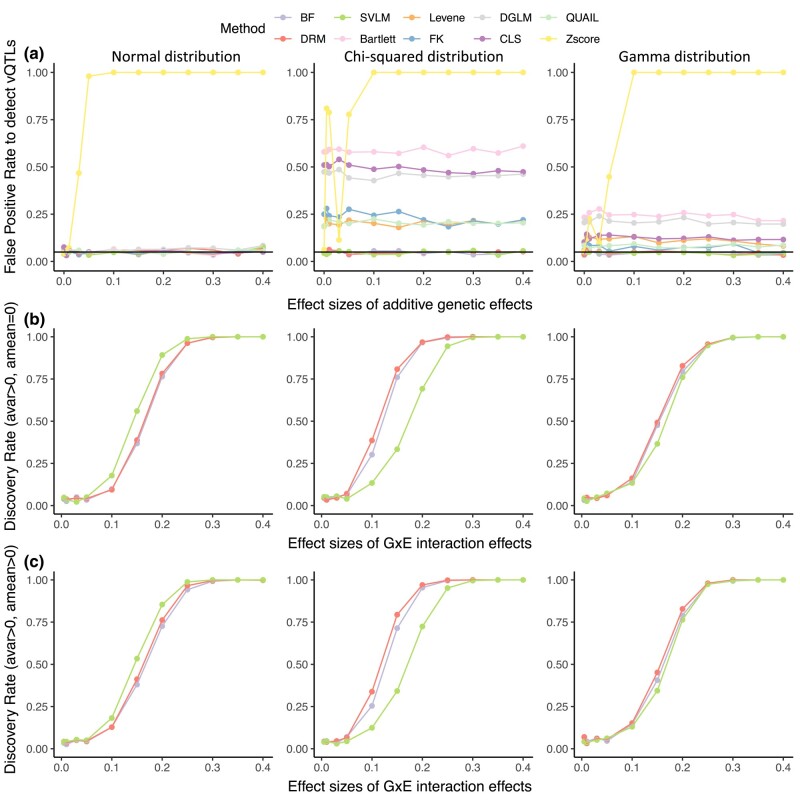
Method performance for uniformly distributed continuous exposure. The discovery rate of methods under the scenario that a single genetic variant affects a) trait level only (*a_mean_* ≥ 0, *a_var_* = 0), which includes the situation where there is no genetic effect (*a_mean_* = 0, *a_var_* = 0), and b) trait level and variance (*a_mean_* = 0.1, *a_var_* > 0). The black horizontal line corresponds to a rate of 0.05. The trait is also affected by noise and environmental factors. The first, second, and third columns represent errors generated from normal, Chi-squared, and gamma distributions, respectively.

When the exposures follow the uniform distribution, the *Z* score method displays the highest FPR, while the SVLM, BF, and DRM methods keep the FPR near 0.05 (Fig. 3a). SVLM outperforms other methods when the error is normally distributed, and DRM performs the best when the error is non-normally distributed (Fig. 3b). Consistent conclusions are observed when the exposure follows the normal distribution (Supplementary Fig. 4). Similar to the finding that the optimal method is affected by the MAF in binary exposures, BF is the optimal method when the MAF equals 0.2 or more irrespective of environmental factor distributions and error distributions.

In summary, in the presence of a *G×E* interaction with continuous exposure, SVLM, DRM, and BF methods show low FPR. At low MAFs, the SVLM shows the highest discovery rate when the phenotype data follow the normal distribution, and DRM performs the best when the phenotype data is non-normally distributed. BF outperforms the other two methods when the MAF increases.

### vQTL detection for unobserved or genetically associated environmental factors

The simulations so far assume that the interacting environmental factor is observed, but this may not always be the case in many settings. Therefore, we also considered scenarios where the interacting environmental factors may be unobserved. We further extended these to situations where the exposure may also be associated with genotype. We carried out simulations with sample sizes of 1,000 and 200,000, and MAFs of 0.05, 0.1, 0.2, and 0.3 to estimate vQTL methods performance under these additional scenarios.

To estimate vQTL methods' performance under the scenario where *E* is unobserved we used the same simulation design, but this time did not initially regress out the *E* as a covariate for phenotype *Y*. Overall, the majority of methods do not show inflated false positive rates, except the *Z* scores for all distributions, as well as the CLS and FK tests with binary exposures only. In terms of discovery rates, the BF and DRM methods do not perform well, but SVLM is still optimal under the majority of biological scenarios. There is one exception under our simulation settings, when the individuals fall in two classes of a binary exposure at the rate of 10 and 90%, and here the DGLM is the optimal method, followed by Bartlett's test and SVLM. Overall, under the same parameter settings, the discovery rate of the optimal method is lower without regressing E than in situations where we regressed out E first. The reasons for this could be differences in data distributions of the raw phenotype data and phenotype residuals after adjusting *E*.

It is also possible that the exposure is affected by genetic effects, for example, height. To explore if vQTL method performance changes in this situation, we simply assumed that the interacting environmental factor *E*1 equals *E* + *a_mean_G* and repeated the simulations. In this situation the method performance is consistent with the conclusions reached when the environmental factor exposures are independent of genetic effects. Overall, BF, DRM, and SVLM do not show inflated FPRs when the environmental exposure categories are under genetic influence. SVLM and DRM are still the optimal methods when the MAF is small, and BF shows better performance when the MAF increases.

### Phenotype data normalization may induce mean-variance associations across groups

**Fig. 4.**
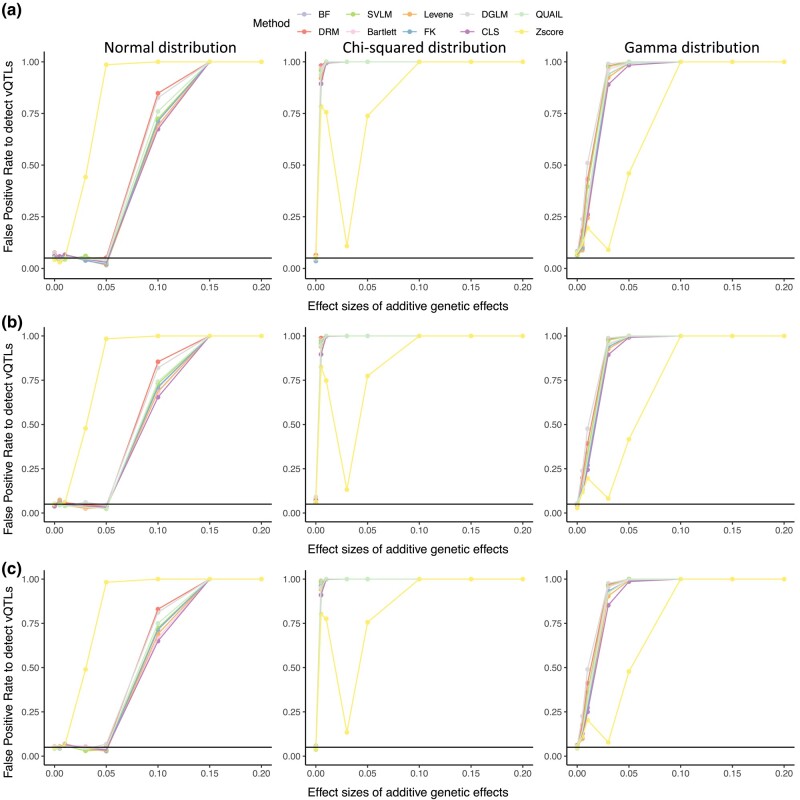
Method performance with rank inverse normal transformed data. Discovery rates of methods under the scenario that a single genetic variant affects trait level only (*a_mean_* ≥ 0, *a_var_* = 0), which includes the situation where there is no genetic effect (*a_mean_* = 0, *a_var_* = 0) when the interacting environmental factor is a) binary factor, b) Uniformly distributed continuous factor and c) Normally distributed continuous factor. The first, second, and third columns represent errors generated from normal, Chi-squared, and gamma distributions, respectively.

Some vQTL detection methods perform better with normally distributed phenotypes, for example, Levene's test, and, therefore, data normalization may improve method performance. However, data transformations can also induce novel mean-variance associations, where the change of phenotype mean level could lead to a change in the variance of the data distribution (Sun *et al*. 2013), for example as observed for the *Z* score regression model (Fig. 2a). To explore this further, we normalized the phenotype data residuals using quantile normalization and repeated the comparison across methods. Taking the binary exposure as an example, all methods showed high FPR for the normalized input data when the error is non-normally distributed (Fig. 4a). When the additive genetic effects increase, the FPRs increase. The same conclusion was observed when the exposure was continuous (Fig. 4b and c). Therefore, we do not recommend data transformations before vQTL identification.

### Power analysis

**Fig. 5.**
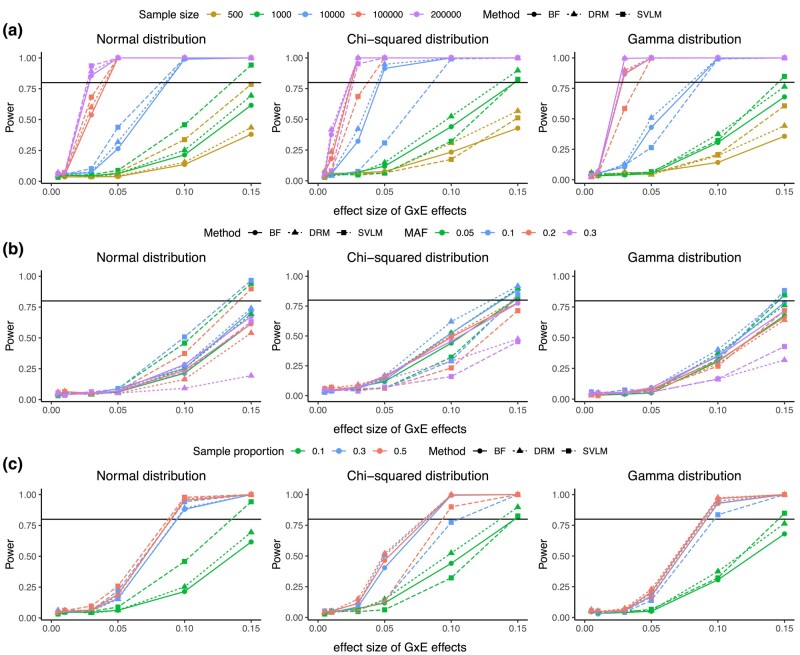
Power analysis when the exposure is binary distributed. Power of BF, SVLM, and DRM across different a) sample sizes, b) MAFs, and c) sample distributions in two exposure categories. The 3 columns represent situations where the phenotype follows the normal (left), Chi-squared (middle), and gamma (right) distributions, respectively.

We estimated the power to detect vQTLs under the scenario where the exposure is binary. When a single genetic variant influences trait variability only (*a_mean_* = 0, *a_var_* > 0), the power of BF, DRM, and SVLM increases with larger sample sizes. For example, with a MAF of 0.05, a sample size of 10,000, and sample proportions in the 2 exposure categories of 10 and 90%, the power of all three methods could reach 80% when the effect size of the GxE interaction is 0.1 (Fig. 5a). If the sample size increases to 200,000, BF, DRM, and SVLM could reach 80% power when the effect size of the vQTL is less than 0.03 (Fig. 5a). As we show for the discovery rates of the methods, the power of each DRM and SVLM are higher than BF with MAF less than 0.2, but the power of BF is the highest when the MAF is 0.3. Furthermore, a more balanced sample distribution across the two exposure categories improves power. Assuming 1,000 individuals and equally distributed samples across the two exposure categories, the power of DRM and SVLM surpasses 80% when the effect size of GxE interaction is near 0.1 (Fig. 5c).

We also estimated the power of the BF, DRM, and SVLM methods when the exposure is continuous (Supplementary Figs. 5 and 6). As expected, a larger sample size improves the power of BF, DRM, and SVLM. When the exposure follows the Uniform distribution, the power of BF, DRM, and SVLM reaches nearly 100% with a sample size of 200,000, 0.05 MAF, and vQTL effect size of 0.05 (Supplementary Fig. 5). When the exposure is normally distributed, with 200,000 samples and a 0.05 MAF, the power of BF, DRM, and SVLM could reach at least 80% for a vQTL effect size of 0.1 (Supplementary Fig. 6).

### Applying DRM, SVLM, and BF to human v-eQTLs

According to our comparison, three approaches showed optimal performance for detecting vQTLs in our simulations, BF, DRM, and SVLM. To further evaluate their ability to identify vQTLs, these three methods were next applied to published data to validate previously detected vQTLs. We analysed RNA-seq gene expression profiles from lymphoblastoid cell lines (LCL) from TwinsUK cohort samples to detect variance expression quantitative trait loci (v-eQTLs) identified by 2 previous studies (Brown *et al*. 2014; Wang, Yang *et al*. 2014). Briefly, Brown *et al*. (2014) applied the CLS method to 765 LCL TwinsUK samples where the gene expression data were generated using RNA-seq; and Wang, Fu *et al*. (2014) (Wang, Yang *et al*. 2014) applied a combination of the FK test and DGLM methods to 825 LCL TwinsUK samples where the gene expression data were generated using Illumina Human HT-12 V3 BeadChips (see Materials and Methods). RNA samples partially overlapped across studies, and both reports identified v-eQTLs in these LCL gene expression datasets. Given that the CLS method from Brown *et al*. (2014), and the FK and DGLM methods from Wang, Fu *et al*. (2014) all showed high false positive rates in our results, we focused only on 5 genes for which both studies detected v-eQTLs, *NUDT2, BTN3A2, CAPN11, PLXDC2,* and *USP6*. We assessed the performance of BF, DRM, and SVLM at these previously reported results.

We obtained and analysed genotype and LCL RNA-seq gene expression profiles from 765 samples from the TwinsUK cohort. The samples, gene expression data, and imputed genotypes are identical to those in Brown *et al*. (2014). In addition to BF, DRM, and SVLM, we also applied the vQTL detection methods that the original studies used (CLS in Brown *et al*. (2014), and a combination of FK and DGLM in Wang, Fu *et al*. (2014) (Wang, Yang *et al*. 2014) to validate the originally published v-eQTL results. Furthermore, both previous studies also reported evidence for epistasis between the identified v-eQTLs and other SNPs (epi-QTLs). Brown *et al*. (2014) explored the SNPs located within 1 Mbp of the transcription start site (TSS) of the target gene, and Wang, Fu *et al*. (2014) (Wang, Yang *et al*. 2014) examined genome-wide SNPs. Overall, we selected 5 genes with 10 v-eQTLs and 12 epi-QTLs for validation using BF, DRM, SVLM, CLS, and the combination of FK and DGLM. Multiple testing was taken into account using permutations.

After permutations, we validated 9 of the 10 previously reported v-eQTLs for these 5 genes using BF, DRM, and SVLM (Table 2). The previously reported v-eQTL rs11011705 (v-eQTL for *PLXDC2* in Brown *et al*. (2014)) did not validate by either method. We also analysed the dataset using the original methods applied by both studies (CLS, FK, DGLM). Altogether, 4 of the 5 v-eQTLs from Brown *et al*. (2014) were detected at nominal significance using CLS, and 3 surpassed the multiple testing threshold. All 5 of v-eQTLs reported by Wang, Fu *et al*. (2014) were validated using DGLM and FK after correcting for multiple testing (FDR < 0.05).

We next explored the performance of 12 SNPs previously reported by Brown *et al*. (2014) and Wang, Fu *et al*. (2014) to interact with v-eQTLs, that is, 12 epi-QTLs (Table 2). We identified 10 of these 12 epi-QTLs as v-eQTLs in our dataset by either BF, DRM, or SVLM. Two SNPs, rs11594747 (epi-QTL for *PLXDC2* in Brown *et al*. (2014)) and rs7032924 (epi-QTL for *NUDT2* in Wang, Fu *et al*. (2014)), did not validate by any method. However, neither of the 2 epi-QTLs was validated when applying the original studies' methods either (CLS, FK, and DGLM).

Overall, 19 of the 22 tested QTLs (vQTLs or epi-QTLs) were successfully validated by BF, SVLM, or DRM after multiple testing corrections. This result shows that the BF, DRM, and SVLM methods can validate the majority of previously detected vQTLs. In terms of validation of epi-QTLs, our results also show that BF, DRM, and SVLM have greater power than CLS to detect interacting QTLs as vQTLs. Besides, 6 of the 22 tested QTLs could not be validated by CLS but were validated by at least 3 other methods, which is consistent with our simulation results that CLS shows a lower discovery rate (Fig. 2b and c). Furthermore, 2 of the 22 tested QTLs were not identified by SVLM but were detected by DRM, FK, and DGLM (rs732090 and rs7746199). Although the FK method also shows good performance in this dataset, the FK method has a high FPR in our simulation, suggesting that it is not the optimal approach in this scenario. In summary, the BF, DRM, and SVLM could successfully detect the majority of previously published v-eQTLs and epi-QTLs.

## Discussion

vQTLs identification could shed light on the genetic basis underlying complex traits. Although multiple approaches have been developed to detect vQTLs, a comprehensive method comparison is lacking. In our study, we used simulated data to evaluate the performance of 10 vQTL detection methods under different biological scenarios of gene-environment interaction. When the environmental exposure is discrete, DRM and SVLM perform well at small MAFs, while BF is optimal at larger MAF. When the environmental exposure is continuous, DRM is optimal in applications to non-normally distributed data, and SVLM is optimal for Normally distributed phenotypes at lower MAF, while BF outperforms the other methods when the MAF is high. In general, the BF, DRM, and SVLM methods display an FPR near 0.05 and perform optimally overall, with differences in performance attributed to data distribution, sample size, and MAF. A larger sample size and more balanced sample distribution in different exposure categories could improve the performance of BF, SVLM, and DRM.

The BF method has often been used to identify vQTL to date (Table 1). However, fewer vQTL studies use SVLM and DRM. The reason might be that to apply SVLM to a dataset one needs to first regress out the genotype from the phenotype as a covariate. Therefore, when undertaking genome-wide vQTL detection for one trait, the genotype adjustment will be performed millions of times, which brings a large computational burden. DRM is relatively novel, and so it has not been used in many studies.

The other seven methods considered in this study, including Levene's test, Bartlett's test, FK test, DGLM, CLS, QUAIL, and *Z* score methods showed high FPR when the data followed a non-normal distribution (Figs. 2 and 3). The *Z* score regression method even showed FPR near 1 when the data are normally distributed. The reason may be that the transformation of the trait to *Z* scores changes the scale and distribution of original data (Shen and Carlborg 2013; Sun *et al*. 2013). The data transformation is highly likely to induce a mean-variance association, leading to an increased FPR.

Our simulation results are consistent with previous comparisons under the assumption of *G×E* interactions. Wang *et al*. (2019) compared the performance of Bartlett's, BF, FK, and DGLM methods, and suggested that the BF test outperforms other methods and that any type of data transformation including using squared, cubic, log, or rank inverse normal transformed data would increase the FPR. Marderstein *et al*. (2021) compared 9 methods (DRM, Levene's test, generalized Levene's test, BF test, Bartlett's test, FK test, DGLM, *Z* score, and SVLM) when sample proportions across discrete exposures are almost balanced. They proposed that the DRM and SVLM show low FPR, and DRM is optimal, but they ignored the situation where the sample proportions in different exposure categories are unbalanced. Miao *et al*. (2022) evaluated 4 methods including HLMM, QUAIL, Levene's test, and DRM. They proposed that the HLMM shows inflated FPR, while QUAIL, BF, and DRM methods are good at controlling the FPR. We have validated their conclusion with their original simulation settings, but the FPRs of QUAIL is high when the trait distribution is largely skewed [50]. Altogether, our results are in line with those obtained from previous studies and also provide a more detailed comparison under multiple biological scenarios.

In our comparison, we excluded the LRT_MV_, HLMM, BTH, and D test. The original code of LRT_MV_ is missing, HLMM failed to converge when the input data is small, and the BTH and D test require permutation tests. Furthermore, similar to HLMM, the LRT_MV_ calculates the likelihood of genetic effects on the trait level, variance, both, or neither to determine how the genetic variant affects the trait distribution. Therefore, like the HLMM, the LRT_MV_ may also fail to converge when the input data is small. Dumitrascu *et al*. (2019) suggested that the BTH shows similar performance to that of DGLM, and its type I error is lowest in comparison to CLS, Levene's test, BF, and DGLM tests when the error follows the Gaussian distribution. However, one assumption of the BTH is that the error follows the Gaussian distribution, while its FPR has not been explored for non-normally distributed traits. The D test compares the accumulated quantile difference of each genotype group. One potential issue is when the trait is largely skewed and the sample size in the 3 genotype groups is unbalanced. Therefore, the accumulated difference is likely to be different and the FPR might be inflated, although the following permutation tests may help to reduce this inflated error.

We applied the BF, DRM, and SVLM methods to validate v-eQTLs from Brown *et al*. (2014) and Wang, Fu *et al*. (2014) (Wang, Yang *et al*. 2014). The samples, genotype version, and gene expression data were the same as those used in Brown *et al*. (2014), but different from those used in Wang, Fu *et al*. (2014) (Wang, Yang *et al*. 2014). Altogether, 3 of 5 v-eQTLs and 4 of 9 epi-QTLs detected by Brown *et al*. (2014) could be validated by CLS in our study, while 4 of 5 v-eQTLs, and 8 of 9 epi-QTLs could be validated by either BF, DRM or SVLM. All 5 v-eQTLs and 2 of the 3 epi-QTL detected by Wang, Fu *et al*. (2014) could be validated by FK, DGLM, BF, DRM, and SVLM methods. One previously reported v-eQTL rs11011705 [v-eQTL for *PLXDC2* in Brown *et al*. (2014)] did not validate. One potential reason for this discrepancy could be the different number of permutations used to control for multiple testing, where Brown *et al*. (2014) used 5 and the current study is based on 20 permutations (for each method, including BF, DRM, SVLM, CLS, FK, and DGLM). The lower number of permutations may introduce spurious signals. Besides, 2 epi-QTLs rs11594747 [epi-QTL for PLXDC2 in Brown *et al*. (2014)] and rs7032924 [epi-QTL for NUDT2 in Wang, Fu *et al*. (2014)] did not validate by either method. However, neither of the interaction terms is significant when we applied the linear model to test for the interaction effect, that is, gene ∼ v-eQTL + epi-QTL + v-eQTL*epi-QTL. Therefore, the putative epistasis interaction effects may be false positives. Another reason could be that there are differences in how the gene expression data were processed in previous studies and this study. Wang, Fu *et al*. (2014) used the coefficient of variation of expression, Brown *et al*. (2014) used the quantile normalized expression data, and this study used the raw reads per kilobase per million mapped reads (RPKM) data. A related reason is differences in the covariates used in the three studies. Wang, Fu *et al*. (2014) considered age and batch effects, while Brown *et al*. (2014) considered 50 latent factors from PEER (probabilistic estimation of expression residuals) (Stegle *et al*. 2012), age and BMI. In contrast, the current study used age, BMI, GC mean, batch effect, zygosity, and family relatedness. The FK method performed well in general and was able to validate signals from Brown *et al*. (2014) as well, however, its FPR is near 0.25 when the error is Chi-squared distributed in our simulations. Altogether, the BF, DRM, and SVLM methods could validate the previously reported v-eQTLs and epi-QTLs.

In our simulation design, the rationale for applying the min–max normalization of *Y* is to reduce bias from encoding environmental factors in the vQTL-only scenario. For example, we encoded the environmental factor as 9 and −1 when the sample proportions in the two exposures were 10 and 90%, and we encoded them as 1 and −1 when the sample proportions were 50 and 50%. For the continuous environmental factors, our exposures follow either a Normal distribution with a mean of 25, or a uniform distribution from 20 to 70. The setting that the sum of *a_mean_, a_E_, a_var_,* and *a_error_* is set to 1 could be a limitation in our study design, and future work can explore the effect of removing this restriction.

There are multiple considerations in applications to identify genome-wide vQTLs. One hurdle is how to efficiently conduct vQTL identification of biological omics data across the genome. Available vQTL studies of gene expression or DNA methylation all focus on genetic variants within certain genomic regions (for example, restricting analyses to local genetic effects) rather than the whole genome (Paré *et al*. 2010; Brown *et al*. 2014; Wang, Yang *et al*. 2014; Ek *et al*. 2018; Sarkar *et al*. 2019). Unlike the identification of mean-controlling QTLs, where linear models are fitted and multiple software are available, many of the vQTL models include additional data analysis steps before vQTL identification. For example, SVLM regresses out the genotype before vQTL detection, DRM and BF calculate the distance of each phenotype data point to a quantile per genotype group. These additional steps can introduce extra computational burden and require additional storage. This is further important because a large sample size is needed to achieve good power for vQTL detection. Given that, the computational time and data storage are challenges in identifying genome-wide vQTLs in large samples.

When interpreting detected signals, it is important to distinguish the true vQTLs from spurious signals. One issue that can impact this is the mean-variance association. The mean level and variance of the data are correlated in data distributions, and this may lead to mean-controlling QTLs being identified as spurious vQTLs. Data transformation is one source of inducing novel mean-variance association because it changes the scale of the phenotype. Therefore, we do not recommend data transformation before vQTL detection. Further considerations include linkage disequilibrium (LD) (Wei *et al*. 2013; Wood *et al*. 2014; Forsberg *et al*. 2015; de los Campos e*t al*. 2019) between the v-QTL and mean-controlling QTLs. A trait controlled by a mean-controlling QTL (SNP_m_) will display different phenotype levels in each genotype group. If a genetic variant (SNP_1_) is in LD with the SNP_m_, it is possible that the phenotypic variance will be different across genotype groups of SNP_1_ (Supplementary Fig. 7) (Hemani *et al*. 2021). To circumvent this, it would be useful to check LD between known mean-controlling QTLs and detected vQTL. However, it is possible that there are hidden mean-controlling QTLs. Another issue that may induce spurious vQTLs is parent-of-origin or imprinted QTLs. In a parent-of-origin model with a single locus, the phenotypes of the *Aa* genotype category depend on whether the *A* allele is inherited from the father or the mother (Lawson *et al.* 2013; Mozaffari *et al*. 2019). Therefore, the *Aa* genotype group will display more phenotypic variability than either *AA* or *aa* genotype group in the population. If either homozygote group is missing or has a small sample size, this genetic variant could be detected as a spurious vQTL. Therefore, we suggest setting a minimum threshold for the sample size in the minor homozygote genotype group in real data applications.

In conclusion, we carried out a simulation study to evaluate the performance of ten methods to detect gene-environment interactions as vQTLs. We observed that the BF, DRM, and SVLM methods are optimal overall, and can validate previously detected vQTLs in human gene expression data. We do not recommend data transformation for vQTL detection, because such a transformation may induce a mean-variance association. To improve the power to detect vQTLs, a larger sample size and more balanced sample distribution across exposure categories are helpful. We also suggest follow-ups including LD estimation and comparison with mean-controlling QTLs in the interpretation of vQTLs identified in real data applications.

## Supplementary Material

jkae022_Supplementary_Data

## Data Availability

The code for carrying out the simulation study and analysis is available at https://github.com/ZXiaopu/vQTL_sim. Access to TwinsUK data can be applied through the cohort data access committee, see https://twinsuk.ac.uk/resources-for-researchers/access-our-data/. Supplemental material available at G3 online.
