# Supplementary material for: Detecting genetic effects on phenotype variability to capture gene-by-environment interactions: a systematic method comparison
Source: G3 (Bethesda). 2024 Jan 30;14(4):jkae022. doi: 10.1093/g3journal/jkae022 (PMC10989912; doi:10.1093/g3journal/jkae022)
Supplement: jkae022_Supplementary_Data [file jkae022_supplementary_data.zip › Supplemental_Material_Legend_G3-2023-404768.pdf]

Supporting information

Figure S1. False positive rates of QUAIL method under different settings.

Figure S2. An example of the phenotype data distribution in three genotype groups with a sample size of 1000, MAF of 0.3, and sample proportions in binary exposures of 10% and 90%. The Y-axis represents A) the unadjusted trait and B) residuals after regressing out the environmental factor E.

Figure S3. Effect size estimation when the interacting environmental factor is A) binary environmental factor, B) Uniformly distributed continuous factor, and C) Normally distributed environmental factor.

Figure S4. Method performance for Normally distributed continuous exposure. Discovery rate of methods under the scenario that a single genetic variant affects A) trait level only (a_{mean} >= 0, a_{var} = 0), which includes the situation where there is no genetic effect (a_{mean} = 0, a_{var} = 0), and B) trait level and variance (a_{mean} = 0.1, a_{var} > 0). The black horizontal line corresponds to a rate of 0.05. The trait is also affected by noise and environmental factors. The first, second, and third columns represent errors generated from Normal, Chi-squared, and Gamma distributions, respectively.

Figure S5. Power analysis when the exposure is Uniformly distributed. Power of BF, SVLM and DRM across different A) sample sizes and B) MAFs. The three columns represent situations

where the phenotype follows the Normal (left), Chi-squared (middle), and Gamma (right) distributions, respectively.

Figure S6. Power analysis when the exposure is Normally distributed. Power of BF, SVLM and DRM across different A) sample sizes and B) MAFs. The three columns represent situations where the phenotype follows the Normal (left), Chi-squared (middle), and Gamma (right) distributions, respectively.

Figure S7. An example of linkage disequilibrium between mean-controlling QTL and vQTL leads to spurious vQTL detection.

Table S1. The optimal method(s) under different sample size and MAFs.
